# A dynamic temperature difference control recording system in shallow lake mesocosm

**DOI:** 10.1016/j.mex.2020.100930

**Published:** 2020-05-22

**Authors:** Tao Wang, Jun Xu, Jorge García Molinos, Chao Li, Bowen Hu, Meng Pan, Min Zhang

**Affiliations:** a College of Fisheries, Huazhong Agricultural University, Hubei Provincial Engineering Laboratory for Pond Aquaculture, Freshwater Aquaculture Collaborative Innovation Center of Hubei Province, Wuhan 430070, PR China; b Donghu Experimental Station of Lake Ecosystems, State Key Laboratory of Freshwater Ecology and Biotechnology of China, Institute of Hydrobiology, Chinese Academy of Sciences, Wuhan 430072, PR China; c Arctic Research Center, Hokkaido University, Sapporo 001-0021, Japan; d Global Station for Arctic Research, Global Institution for Collaborative Research and Education, Hokkaido University, Sapporo 001-0021, Japan; e Graduate School of Environmental Science, Hokkaido University, Sapporo 060-0810, Japan

**Keywords:** Warming, Control experiment, Freshwater

## Abstract

The effects of climate change on shallow lakes were studied via control experiments, such as a mesocosm study. Accurate control, monitoring and recording of temperature difference are crucial for the ongoing simulation of warming mesocosm. In this article, we provide a method that can adjust automatically and allow real-time monitoring and recording of water temperature. This system is composed of three main parts: the temperature sensor DS18B20, which measures and outputs the digital temperature value; a C8051F320 microcontroller, which acquires, analyses and stores the temperature data and performs control upon start and shutdown of external heating elements; and external heating devices perform heating until the target temperature difference is achieved.

• This system can maintain a certain temperature difference under gradually changing external environmental conditions.
• This system can achieve real-time online monitoring of water temperature.
• This system has an excellent ability to resist disturbance.

## Graphical abstract

**Figure:**
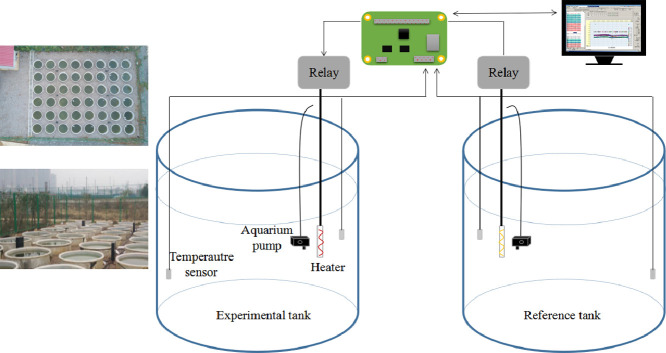

**Specifications Table**

**Table:** 

Subject Area:	Environmental Science
More specific subject area:	Freshwater ecology (mesocosm study)
Method name:	A dynamic temperature difference control recording system
Name and reference of original method:	Huang Y., Xu J., Zhang M. [1]. A dynamic controlling equipment for differental temperature (in Chinese). Chinese Patent No. CN2757476.
Resource availability:	Not appliable

## Method details

### Background

Shallow lakes represent the most abundant type of freshwater ecosystems globally. They support high levels of biodiversity and provide important ecosystem services [2]. They are also particularly exposed and vulnerable to human disturbances. Among the threats to shallow lakes, climate change is a serious emerging threat [3]. Anticipating the consequences of climate change to aquatic ecosystems is complicated because of the idiosyncrasy in response among aquatic species often results in unexpected community effects [4]. A series of studies have investigated the effects of climate change on shallow lakes using different approaches, such as enclosure experiments and analysis of inter-annual fluctuations or differences between various climate regions [5], [6], [7]. Controlled experimental systems are often designed to investigate the effects of climate warming on shallow lakes [8], [9], [10], [11] and accurate control of the temperature difference between warming and reference groups is crucial to ensuring the veracity of experimental results. In the study, we introduce a dynamic recording system for controlling temperature difference that can be used in mesocosms study.

### Description

**Fig. 1:**
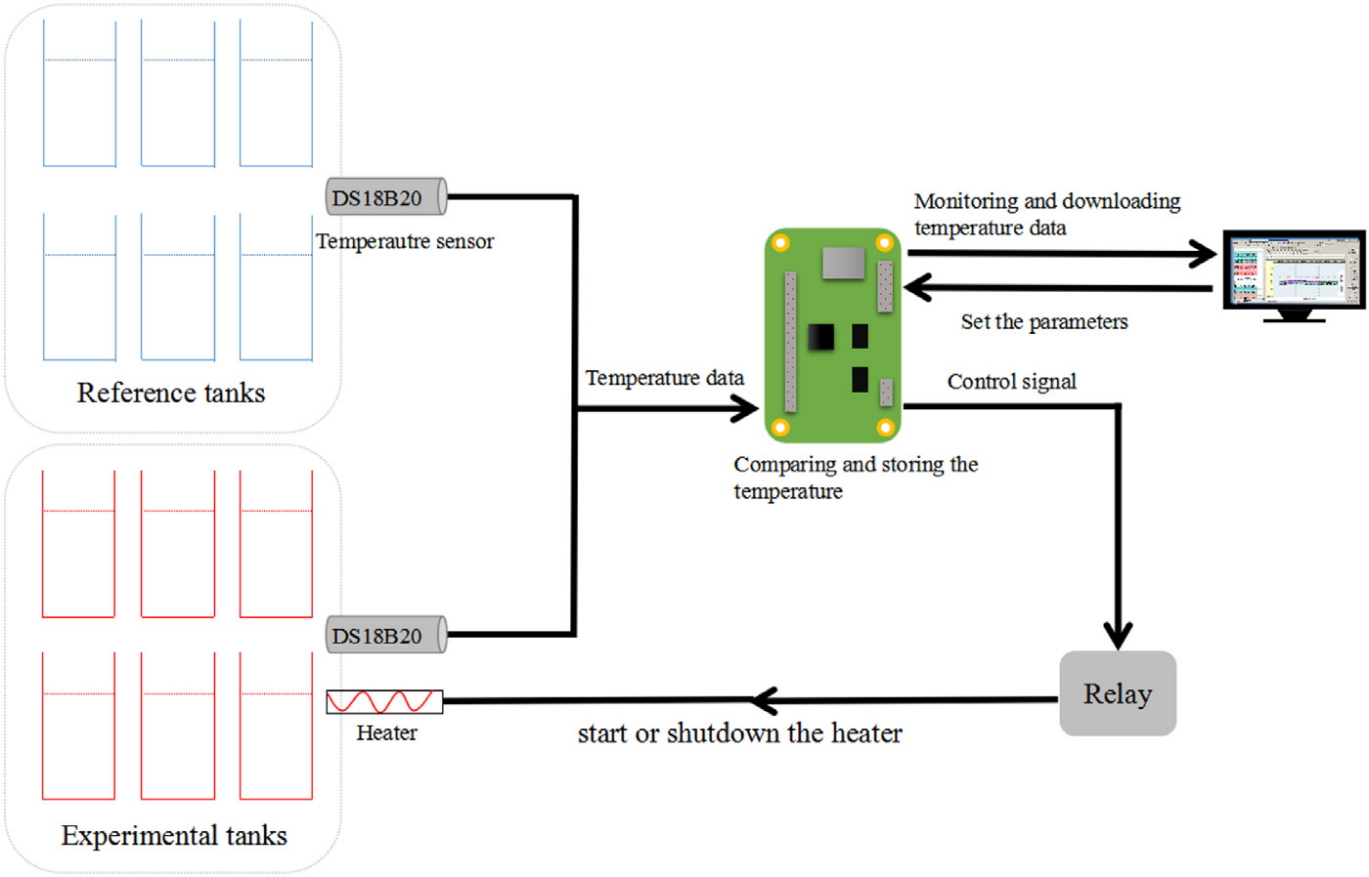
Schematic representation of the system.

This recording system for controlling temperature difference can control heat devices for experiments automatically and allow real-time monitoring and recording of water temperature. The system is composed of temperature sensors, heating devices and a controller. The temperature sensor of the experimental group is used to detect the water temperature of the heating mesocosm, while the temperature sensor of the reference group is utilised to detect the water temperature of the ambient condition. The experimental and reference groups are subjected to the same external environmental conditions. The output terminals of the temperature sensors for the experimental and reference groups are connected to the input terminals of the controller, the output terminal of which is connected to the heater of the experimental group. The controller is used to acquire temperature data from the temperature sensors of the experimental and reference groups. The start and shutdown of the experimental group heater, which is used to heat the water temperature, are controlled after comparing the temperature data (Fig. 1).

This system can maintain a certain temperature difference between the experimental and reference groups (external environment) under gradually changing external environmental conditions. The reference group is not heated by the heater to maintain the temperature to similar to that of the external environment. This system has an excellent ability to resist disturbance, allowing it to improve the accuracy of experimental temperature control conditions and perform efficiently.

### Building and deploying

The temperature sensors of the experimental and reference groups described above are the digital temperature sensor DS18B20 (Maxim IC, USA), which can output the digital temperature value directly. DS18B20 measures the temperature range from −55°C to 125°C with a ±0.5°C accuracy range from −10°C to 85°C (https://www.maximintegrated.com/en/products/sensors/DS18B20.html). Digital communication has a remarkable capacity to resist interference because the 1-Wire communication protocol adopted for DS18B20 has excellent CRC fault-tolerant mechanism, which has the advantages of strong anti-interference ability and reliability of communication and immunity from fault data, thereby preventing the acquisition system from storing faulty temperature values and improving the reliability of the monitoring system. Temperature sensors were located 0.5 m below the water surface in both unheated and heated mesocosm. In order to improve the accuracy of temperature measurement, another temperature sensor can be added on the other side of per mesocosm.

The controller described above is a C8051F320 (Silicon Labs, USA) microcontroller that can perform sampling through the externally connected temperature sensors of the experimental and reference groups. Controlling via an external heater (A10-3, Xin Shao Guang, China) can be programmed into the chip through a programming unit. The J-JTAG, which is the interface of the programming unit, is used to burn the written user program into the C8051F320 microcontroller. The SW-Reset part provides correct timing of power upon reset for the controller. A Darlington transistor array is installed between the controller and the external heater. One end of the Darlington transistor array is connected to the output terminal of the controller and the other end is connected to the experimental group heater. The transistor array can amplify the digital output signal of the controller, producing a control signal of the relay to start or shutdown the heater.

The C8051F320 microcontroller can acquire the temperature from digital temperature sensors through the port P20–P27 and analyse the data. Based on the analysis and processing results, control upon start and shutdown of the experimental group heater is performed. At the same time, the temperature data are stored in the FLASH at certain intervals. The controller also includes a Mini-B connector of the USB interface, which is used to interconnect with the computer to set the parameters of the controller and download the sampling data as well as monitor the on-line temperature in real-time.

The controller described above mainly performs the following controls: Firstly, we set the temperature difference between the experimental and the reference group (Tdx). Secondly, after the controller acquires the temperature data from the temperature sensors of the experimental and reference groups, the average temperature from the reference group sensor (Trm) is compared with that of the experimental group sensor (Tci). If Tci - Trm < Tdx, the experimental group heater corresponding to the experimental group sensor is started to perform heating; otherwise, the corresponding heater is shut down. When one tank in the experimental group is installed with several temperature sensors, Tci will become the average value of temperatures from these sensors.

### Method validation

**Fig. 2:**
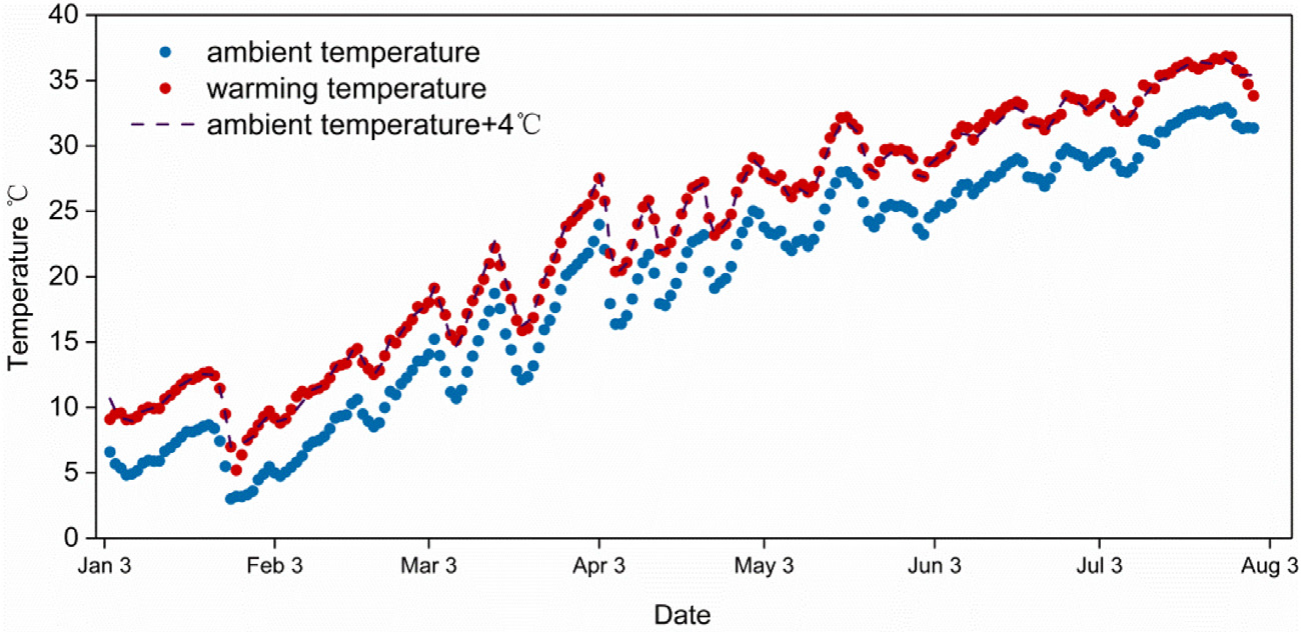
Mean daily water temperature in the mesocosms. Ambient temperature represent average water temperature in the unheated mesocosms, warming temperature represent average water temperature in the heated mesocosms, we design a 4°C temperature difference between them, error bars indicate ± standard error, n=12 replicates per treatment. Ambient temperature + 4°C represent the design values of warming temperature.

This system have been used in our previous studies [8,9,12]. As shown in Fig. 2, We set up a simulated warming group which involving a sustained increase in temperature of 4°C above control ambient conditions by this temperature difference controll recording system [see 13]. Water temperature in the mesocosms followed the desired design.
